# Possibility of avoiding axillary lymph node dissection by immune microenvironment monitoring in preoperative chemotherapy for breast cancer

**DOI:** 10.1186/s12967-018-1692-3

**Published:** 2018-11-19

**Authors:** Koji Takada, Shinichiro Kashiwagi, Wataru Goto, Yuka Asano, Katsuyuki Takahashi, Hisakazu Fujita, Tsutomu Takashima, Shuhei Tomita, Kosei Hirakawa, Masaichi Ohira

**Affiliations:** 1 Department of Surgical Oncology, Osaka City University Graduate School of Medicine, 1-4-3 Asahi-machi, Abeno-ku, Osaka, 545-8585 Japan; 2 Department of Pharmacology, Osaka City University Graduate School of Medicine, 1-4-3 Asahi-machi, Abeno-ku, Osaka, 545-8585 Japan; 3 Department of Scientific and Linguistic Fundamentals of Nursing, Osaka City University Graduate School of Nursing, 1-5-17 Asahi-machi, Abeno-ku, Osaka, 545-0051 Japan

**Keywords:** Sentinel lymph node biopsy, Breast cancer, Microenvironment, Neoadjuvant chemotherapy, Tumor-infiltrating lymphocytes

## Abstract

**Background:**

The diagnosis of metastasis by sentinel lymph node biopsy (SLNB) in early breast cancer surgery provides an accurate view of the state of metastases to the axillary lymph nodes, and it has now become the standard procedure. In the present study, whether omission of axillary lymph node dissection (ALND) after neoadjuvant chemotherapy (NAC) is possible by evaluation of tumor-infiltrating lymphocytes (TILs) before NAC in cases without metastasis on diagnostic imaging, but with metastasis on SLNB, was retrospectively investigated.

**Methods:**

A total of 91 patients with resectable, early-stage breast cancer, diagnosed as cT1–2, N0, M0, underwent SLNB and were treated with NAC. A semi-quantitative evaluation of lymphocytes infiltrating the peritumoral stroma as TILs in biopsy specimens of primary tumors prior to treatment was conducted.

**Results:**

In cases with a low number of TILs, estrogen receptor expression was significantly higher (p = 0.044), and human epidermal growth factor receptor 2 (HER2) expression was significantly lower than in other cases (p = 0.019). The number of TILs was significantly lower in cases in which the intrinsic subtype was hormone receptor-positive breast cancer (HRBC) (p = 0.044). Metastasis to axillary lymph nodes was significantly more common in HER2-negative cases and cases with a low number of TILs (p = 0.019, p = 0.005, respectively).

**Conclusions:**

Even if macrometastases are found on SLNB in cN0 patients, it appears that ALND could be avoided after NAC in cases with a good immune tumor microenvironment of the primary tumor.

**Electronic supplementary material:**

The online version of this article (10.1186/s12967-018-1692-3) contains supplementary material, which is available to authorized users.

## Background

The diagnosis of metastasis by sentinel lymph node biopsy (SLNB) in early breast cancer surgery (BCS) provides an accurate view of the state of metastasis to the axillary lymph nodes, and it has now become the standard procedure [1–5]. However, in cases requiring neoadjuvant chemotherapy (NAC), it has not yet been established whether SLNB should be done before or after NAC. Performing SLNB after NAC, that is, during surgery, means that there is only one surgery, which reduces the burden on patients, but there is an increased chance of false-negative results. Therefore, our institute performs SLNB before NAC, and then NAC is given based on the histologic diagnosis, and BCS is finally performed [6]. Several reports recommend this method [7–10]. In this method, accurate pathological diagnosis can be performed before NAC, while unnecessary axillary lymph node dissection (ALND) can be avoided if there is no metastasis to axillary lymph nodes. However, if the axillary lymph node metastasis disappears following NAC, unnecessary ALND might be performed.

The immune tumor microenvironment (iTME) in cancer is currently thought to be involved in many antitumor treatment effects, and the presence of tumor-infiltrating lymphocytes (TILs) has been shown to be a useful indicator to monitor [11–13]. Similarly, TILs could be useful for predicting the effect of NAC in breast cancer [14]. However, few reports have examined the use of TILs as biomarkers in clinical practice.

In the present study, we hypothesized that ALND after NAC can be avoided by evaluation of the iTME before NAC. Then, whether omission of ALND after NAC is possible by evaluation of TILs before NAC in cases without metastasis on diagnostic imaging, but with metastasis on SLNB, was retrospectively investigated.

## Methods

### Patient background

A total of 91 patients with resectable, early-stage breast cancer, diagnosed as cT1–2, N0, M0, underwent SLNB and were treated with NAC at Osaka City University Hospital from August 2009 to July 2016. TNM staging was evaluated according to the seventh edition of the American Committee on Cancer staging manual [15]. Breast cancer was diagnosed histologically by core needle biopsy (CNB) or vacuum-assisted biopsy (VAB) and staged with systemic imaging studies, including computed tomography (CT), ultrasonography (US), and bone scintigraphy. Depending on the immunohistochemical expressions of estrogen receptor (ER), progesterone receptor (PgR), human epidermal growth factor receptor 2 (HER2), and Ki67, the breast cancers were categorized into the following immunophenotypes: luminal A (ER+ and/or PgR+, HER2−, Ki67-low); luminal B (ER+ and/or PgR+, HER2+; ER+ and/or PgR+, HER2−, Ki67-high); HER2BC (HER2-enriched breast cancer; ER−, PgR−, and HER2+); and TNBC (triple-negative breast cancer; negative for ER, PgR, and HER2) [16]. In this study, luminal A and luminal B types were considered hormone receptor-positive breast cancer (HRBC). Sentinel lymph nodes (SNs) were identified by a combination of radioisotope and dye methods, for which the detailed methods have been previously reported [6, 17, 18]. Histopathological diagnosis of lymph node metastasis was made by slicing the entire SN into 2-mm-thick sections [19, 20]. A positive diagnosis of SN metastasis as an indication for axillary clearance was defined as macrometastasis in the SN (macrometastasis: tumor diameter > 2 mm). Micrometastasis and isolated tumor cells were considered negative indications for axillary clearance (micrometastasis: tumor diameter > 0.2 mm, ≤ 2 mm or < 200 tumor cells; isolated tumor cells: tumor diameter < 0.2 mm or < 200 tumor cells) [21]. NAC was generally recommended according to the intrinsic subtype of the primary tumor determined from the biopsy sample. ALND was followed by BCS within 4 weeks after the termination of NAC in SN-positive patients, and BCS without ALND was performed in SN-negative patients.

NAC consisted of four courses of FEC100 (500 mg/m^2^ fluorouracil, 100 mg/m^2^ epirubicin, and 500 mg/m^2^ cyclophosphamide) every 3 weeks, followed by 12 courses of 80 mg/m^2^ paclitaxel administered weekly. Patients with HER2BC were additionally given weekly (2 mg/kg) or tri-weekly (6 mg/kg) trastuzumab during paclitaxel treatment [22–24]. Therapeutic anti-tumor effects were evaluated according to the Response Evaluation Criteria in Solid Tumors [25]. Patients underwent mastectomy or breast-conserving surgery following NAC [26]. In all cases with SN macrometastasis, ALND was performed. The pathological effects of chemotherapy were evaluated in primary tumor resected at the time of BCS. A pathological complete response (pCR) was defined as the complete disappearance of the invasive components of the lesion with or without intraductal components, including within the lymph nodes, according to the National Surgical Adjuvant Breast and Bowel Project B-18 protocol [27].

### Histopathological evaluation of TIL status

**Fig. 1:**
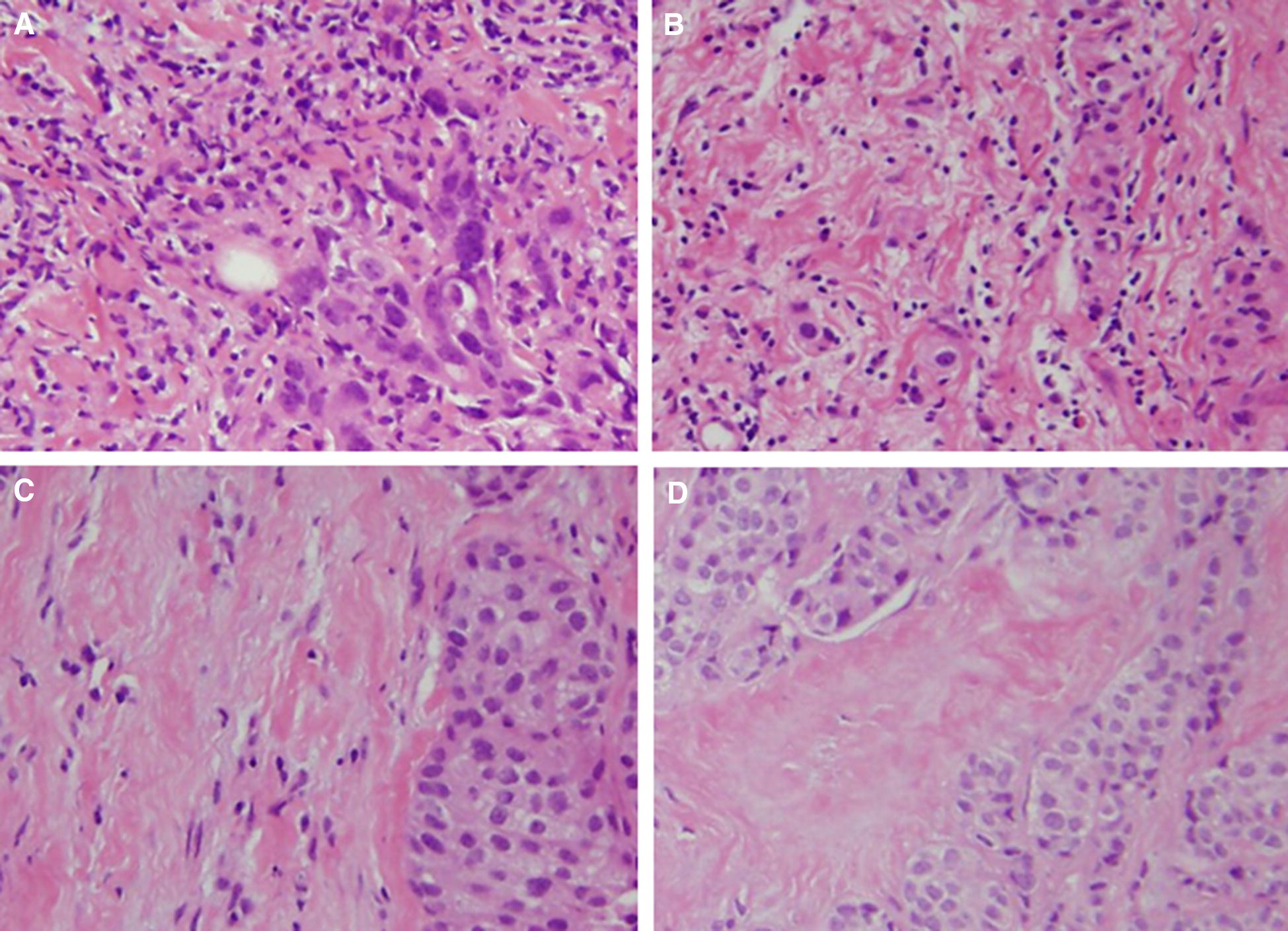
Histopathological evaluation of TILs. TILs were evaluated on biopsy specimens by measuring the percentage of area occupied by lymphocytes on the hematoxylin and eosin (H&E)-stained tumor section at the time of breast cancer diagnosis. The area of the stroma region with lymphoplasmacytic infiltration was > 50%, > 10–50%, ≤ 10%, or absent, and the corresponding score assigned was 3, 2, 1, or 0, respectively (**A**–**D** respectively)

TILs were evaluated on biopsy specimens (CNB or VAB) by measuring the percentage of area occupied by lymphocytes on the hematoxylin and eosin (H&E)-stained tumor section at the time of breast cancer diagnosis [28]. The area of the stroma region with lymphoplasmacytic infiltration was > 50%, > 10–50%, ≤ 10%, or absent, and the corresponding score assigned was 3, 2, 1, or 0, respectively [29] (Fig. 1). TIL status was evaluated as “high” with scores of 2 or more, and “low” with scores of 1 and 0, according to a previous report [29]. The cut-off value of TILs was calculated by receiver operating characteristic (ROC) curve analysis, and the area under the curve (AUC) was 0.719, with a specificity of 0.917 and a sensitivity of 0.750 (Additional file 1: Fig. S1). Histopathological diagnosis was performed by two breast cancer pathologists in blinded fashion.

### Statistical analysis

Statistical analysis was conducted using the JMP software package (SAS, Tokyo, Japan). The relationship between each factor was examined using the Chi squared test (or Fisher’s exact test when necessary). A *p* value < 0.05 was considered significant.

### Ethics statement

This research was conducted at Osaka City University Hospital, Osaka, Japan. Sufficient explanation was provided, and written, informed consent was obtained from all study subjects for their involvement in this study and for the storage and use of their data. This study conformed to the provisions of the Declaration of Helsinki (2013). The study protocol was approved by the Ethics Committee of the Osaka City University (approval number #926).

## Results

### Statistical data of cases that underwent SLNB before NAC and ALND at the time of breast cancer surgery

**Fig. 2:**
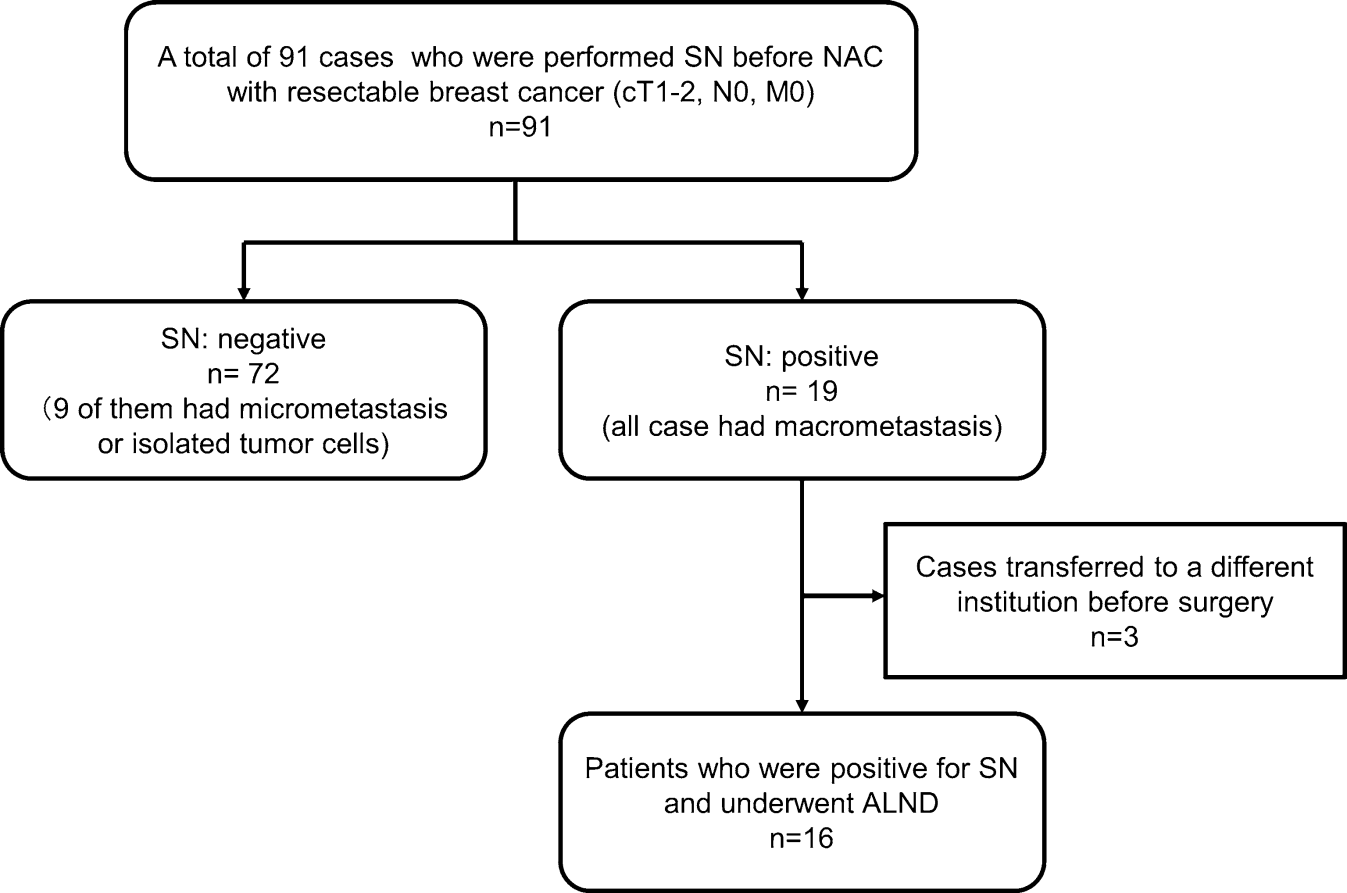
Consort diagram. A total of 91 patients with resectable, early-stage breast cancer, diagnosed as cT1–2, N0, M0, underwent sentinel lymph node biopsy and were treated with NAC. Nineteen (20.9%) of 91 patients who underwent sentinel lymph node biopsy before NAC had metastasis, and three of them were transferred to a different institution before surgery. Thus, 16 cases underwent axillary lymph node dissection at the time of breast cancer surgery

Nineteen (20.9%) of 91 patients who underwent SLNB before NAC had metastasis, and three of them were transferred to a different institution before surgery. Thus, 16 cases underwent ALND at the time of BCS (Fig. 2). All patients were women, with a median age of 47 years (range 28–72 years). The median tumor size was 25.1 mm (range 18.9–42.0 mm). Regarding intrinsic subtypes, nine cases (56.3%) were HRBC, four (25.0%) were HER2BC, and three (18.7%) were TNBC. Twelve cases (75.0%) had a high number of TILs, and four cases (25.0%) had a low number of TILs at diagnosis of breast cancer. In 13 cases (81.3%), multiple SNs were removed at the time of SLNB. In 12 cases (75.0%), metastasis was found in only one of the SNs. The median metastatic diameter was 3397 µm (range 2108–7281 µm). All cases responded to NAC, and the pCR rate was 31.2%. There were four cases (25.0%) in which metastasis was observed in the axillary lymph node on ALND (Table 1).

**Table 1:** Statistical data of 16 patients who underwent SNLB before NAC and ALND at the time of breast cancer surgery

Parameters (*n *= 16)	Number of patients (%)
Age (years old)	47 (28–72)
Tumor size (mm)	25.1 (18.9–42.0)
Estrogen receptor	
Negative/positive	7 (43.8%)/9 (56.2%)
Progesterone receptor	
Negative/positive	10 (62.5%)/6 (37.5%)
HER2	
Negative/positive	8 (50.0%)/8 (50.0%)
Ki67	
Negative/positive	4 (25.0%)/12 (75.0%)
Intrinsic subtype	
HRBC/HER2BC/TNBC	9 (56.3%)/4 (25.0%)/3 (18.7%)
Tumor-infiltrating lymphocytes	
Low/high	4 (25.0%)/12 (75.0%)
Number of excised sentinel lymph nodes	
1/2/3	3 (18.7%)/6 (37.5%)/7 (43.8%)
Number of sentinel lymph nodes with metastasis	
1/2/3	12 (75.0%)/1 (6.3%)/3 (18.7%)
Size of sentinel lymph node itself (mm)	12.5 (9.1–26.3)
Size of metastatic lesion (μm)	3397 (2108–7281)
Clinical response	
cPR/cCR	14 (87.5%)/2 (12.5%)
Pathological complete response	
pCR/non-pCR	5 (31.2%)/11 (68.8%)
Number of lymph node dissection	9 (3–24)
Lymph node metastasis	
Negative/positive	12 (75.0%)/4 (25.0%)
Number of lymph node metastasis	
1/2/6	2 (12.5%)/1 (6.3%)/1 (6.3%)

*SLNB* sentinel lymph node biopsy, *NAC* neoadjuvant chemotherapy, *ALND* axillary lymph node dissection, *BCS* breast cancer surgery, *HER2* human epidermal growth factor receptor 2, *HRBC* hormone receptor-positive breast cancer, *HER2BC* HER2-enriched breast cancer, *TNBC* triple negative breast cancer, *cPR* clinical partial response, *cCR* clinical complete response, *pCR* pathological complete response

### Correlations between clinicopathological features and number of TILs

In cases with a low number of TILs, ER expression was significantly higher (p = 0.044), and HER2 expression was significantly lower than in other cases (p = 0.019). The number of TILs was significantly lower in cases in which the intrinsic subtype was HRBC (p = 0.044). There was no correlation between other clinicopathological features and the number of TILs (Table 2).

**Table 2:** Correlations between clinicopathological features and the number of TILs

Parameters	TILs		*p* value
	High (*n* = 12)	Low (*n* = 4)	
Age			
≤ 47	7 (58.3%)	1 (25.0%)	
> 47	5 (41.7%)	3 (75.0%)	0.278
Tumor size			
≤ 25	6 (50.0%)	2 (50.0%)	
> 25	6 (50.0%)	2 (50.0%)	1.000
Estrogen receptor			
Negative	7 (58.3%)	0 (0.0%)	
Positive	5 (41.7%)	4 (100.0%)	0.044
Progesterone receptor			
Negative	9 (75.0%)	1 (25.0%)	
Positive	3 (25.0%)	3 (75.0%)	0.082
HER2			
Negative	4 (33.3%)	4 (100.0%)	
Positive	8 (66.7%)	0 (0.0%)	0.019
Ki67			
Negative	2 (16.7%)	2 (50.0%)	
Positive	10 (83.3%)	2 (50.0%)	0.207
Intrinsic subtype HRBC			
Non-HRBC	7 (58.3%)	0 (0.0%)	
HRBC	5 (41.7%)	4 (100.0%)	0.044
Intrinsic subtype HER2BC			
Non-HER2BC	8 (66.7%)	4 (100.0%)	
HER2BC	4 (33.3%)	0 (0.0%)	0.207
Intrinsic subtype TNBC			
Non-TNBC	9 (75.0%)	4 (100.0%)	
TNBC	3 (25.0%)	0 (0.0%)	0.298
Number of sentinel lymph nodes with metastasis			
1, 2	10 (83.3%)	3 (75.0%)	
3	2 (16.7%)	1 (25.0%)	0.734
Size of sentinel lymph node itself (mm)			
≤ 12.5	6 (50.0%)	2 (50.0%)	
> 12.5	6 (50.0%)	2 (50.0%)	1.000
Size of metastatic lesion			
≤ 3400	5 (41.7%)	3 (75.0%)	
> 3400	7 (58.3%)	1 (25.0%)	0.278
Clinical response			
cPR	10 (83.3%)	4 (100.0%)	
cCR	2 (16.7%)	0 (0.0%)	0.417
Pathological complete response			
Non-pCR	7 (58.3%)	4 (100.0%)	
pCR	5 (41.7%)	0 (0.0%)	0.136

*TILs* tumor-infiltrating lymphocytes, *HER2* human epidermal growth factor receptor 2, *HRBC* hormone receptor-positive breast cancer, *HER2BC* HER2-enriched breast cancer, *TNBC* triple negative breast cancer, *cPR* clinical partial response, *cCR* clinical complete response, *pCR* pathological complete response

### Correlations between clinicopathological features and metastasis to axillary lymph nodes

Metastasis to axillary lymph nodes was significantly more common in HER2-negative cases and cases with a low number of TILs (p = 0.019, p = 0.005, respectively). However, no correlations were found between other clinicopathological features and axillary lymph node metastasis (Table 3).

**Table 3:** Correlations between clinicopathological features and axillary lymph node metastasis

Parameters	Axillary lymph node		*p* value
	Negative (*n* = 12)	Positive (*n* = 4)	
Age			
≤ 47	6 (50.0%)	2 (50.0%)	
> 47	6 (50.0%)	2 (50.0%)	1.000
Tumor size			
≤ 25	7 (58.3%)	1 (25.0%)	
> 25	5 (41.7%)	3 (75.0%)	0.278
Estrogen receptor			
Negative	6 (50.0%)	1 (25.0%)	
Positive	6 (50.0%)	3 (75.0%)	0.417
Progesterone receptor			
Negative	8 (66.7%)	2 (50.0%)	
Positive	4 (33.3%)	2 (50.0%)	0.582
HER2			
Negative	4 (33.3%)	4 (100.0%)	
Positive	8 (66.7%)	0 (0.0%)	0.019
Ki67			
Negative	3 (25.0%)	1 (25.0%)	
Positive	9 (75.0%)	3 (75.0%)	1.000
Intrinsic subtype HRBC			
Non-HRBC	6 (50.0%)	1 (25.0%)	
HRBC	6 (50.0%)	3 (75.0%)	0.417
Intrinsic subtype HER2BC			
Non-HER2BC	8 (66.7%)	4 (100.0%)	
HER2BC	4 (33.3%)	0 (0.0%)	0.207
Intrinsic subtype TNBC			
Non-TNBC	10 (83.3%)	3 (75.0%)	
TNBC	2 (16.7%)	1 (25.0%)	0.734
TILs			
Low	1 (8.3%)	3 (75.0%)	
High	11 (91.7%)	1 (25.0%)	0.005
Number of sentinel lymph nodes with metastasis			
1, 2	11 (91.7%)	2 (50.0%)	
3	1 (8.3%)	2 (50.0%)	0.071
Size of sentinel lymph node itself (mm)			
≤ 12.5	6 (50.0%)	2 (50.0%)	
> 12.5	6 (50.0%)	2 (50.0%)	1.000
Size of metastatic lesion			
≤ 3400	6 (50.0%)	2 (50.0%)	
> 3400	6 (50.0%)	2 (50.0%)	1.000
Clinical response			
cPR	10 (83.3%)	4 (100.0%)	
cCR	2 (16.7%)	0 (0.0%)	0.417
Pathological complete response			
Non-pCR	8 (66.7%)	3 (75.0%)	
pCR	4 (33.3%)	1 (25.0%)	0.774

*TILs* tumor-infiltrating lymphocytes, *HER2* human epidermal growth factor receptor 2, *HRBC* hormone receptor-positive breast cancer, *HER2BC* HER2-enriched breast cancer, *TNBC* triple negative breast cancer, *cPR* clinical partial response, *cCR* clinical complete response, *pCR* pathological complete response

## Discussion

Metastasis to axillary lymph nodes affects prognosis, so evaluation of axillary lymph nodes is important [30]. Currently, it is recognized clinically that SLNB can accurately diagnose the presence or absence of axillary lymph node metastasis in early-stage breast cancer with no axillary lymph node metastasis [31]. Therefore, if the SN is negative, it is standard practice to omit ALND. On the other hand, NAC is a standard initial treatment not only in locally advanced breast cancer, but also early-stage breast cancer, because it improves the breast conservation rate by downstaging [22, 23, 27]. However, the timing of SLNB in patients undergoing NAC has been debated extensively [32–34]. By performing SLNB after NAC, the state of metastasis to the axillary lymph node at the time of BCS can be known, and the axillary preservation rate is increased; however, the false-negative rate increases [35, 36]. This is caused by lymph flow changes and lymph node scarring due to NAC. The false-negative rate is reported as 11–39% [32–34, 37]. Thus, some studies suggested that SLNB after NAC cannot predict the state of the axillary lymph nodes [38, 39]. Some studies recommend SLNB before NAC [7, 9, 10]. However, with this protocol, while the false-negative rates can be reduced by evaluation with H&E staining, unnecessary lymph node dissection may be performed in cases that are downstaged by NAC. Overall, 20–40% of cN+ cases before NAC will downstage to cN0 after NAC [40, 41]. Evaluation of axillary lymph node metastasis after NAC is difficult in cN0 cases in which metastasis to SNs is observed on pathology.

The iTME in cancer is involved in many antitumor treatment effects [42]. The number of TILs is being established as a biomarker for therapeutic effect and prognosis [11–13]. There are reports that the number of TILs is related to the rate of pCR [14]. In breast cancer, the correlation between subtype and TILs was examined, and it is often reported that it is high in TNBC and HER2BC [43, 44]. In the examination of TILs and clinical factors in the present study, the number of TILs was significantly higher in ER-negative cases than in ER-positive cases, and higher in HER2-positive than in HER2-negative cases; that is, the present result was similar to the previous reports. In the high TILs group, a better therapeutic effect was observed, and remnants of metastases to the axillary lymph nodes were significantly decreased.

When the metastasis to the SN is 2 mm or less, there is little metastasis to lymph nodes that are not the SN, and there are no significant differences in disease-free survival and overall survival between the SLNB alone group and the SLNB with complete ALND group [21, 45–47]. However, if the metastasis is 2 mm or more, half of the patients have metastasis to non-sentinel lymph nodes, and there is a difference in prognosis. Although studies are being conducted to identify other new criteria, there are currently no clinically applicable ones [48]. Although methods for reducing the false-negative rate after NAC have also been studied, many of them require additional examinations or other treatment [49]. However, the present method only requires the examination of H&E-stained specimens, and does not require special examinations or other costly tests.

There are many reports on the scoring of TILs as prognostic factors and effect predictors. However, application to clinical practice has not been reported much. Although the present study is limited by its retrospective nature and the low number of cases studied, it does show the possibility of using TILs as a biomarker in the clinical setting. If this method were established clinically, the disadvantage of SLNB before NAC would be reduced, unnecessary surgery could be avoided, and it would be possible to reduce the burden on patients.

## Conclusions

Even if macrometastases are found in the SN in cN0 patients, it appears that ALND could be avoided if the iTME is good.

## Additional file

**Additional file 1: Fig. S1.** The cut-off value of TILs was calculated by receiver operating characteristic (ROC) curve analysis, and the area under the curve (AUC) was 0.719, with a specificity of 0.917 and a sensitivity of 0.750.

## Abbreviations

SLNB: sentinel lymph node biopsy
BCS: breast cancer surgery
ALND: axillary lymph node dissection
NAC: neoadjuvant chemotherapy
TILs: tumor-infiltrating lymphocytes
ER: estrogen receptor
HER2: human epidermal growth factor receptor 2
HRBC: hormone receptor-positive breast cancer
iTME: immune tumor microenvironment
CNB: core needle biopsy
VAB: vacuum-assisted biopsy
CT: computed tomography
US: ultrasonography
SN: sentinel lymph node
pCR: pathological complete response
H&E: hematoxylin and eosin
