# Supplementary figures and images for: Possibility of avoiding axillary lymph node dissection by immune microenvironment monitoring in preoperative chemotherapy for breast cancer
Source: J Transl Med. 2018 Nov 19;16:318. doi: 10.1186/s12967-018-1692-3 (PMC6245906; doi:10.1186/s12967-018-1692-3)

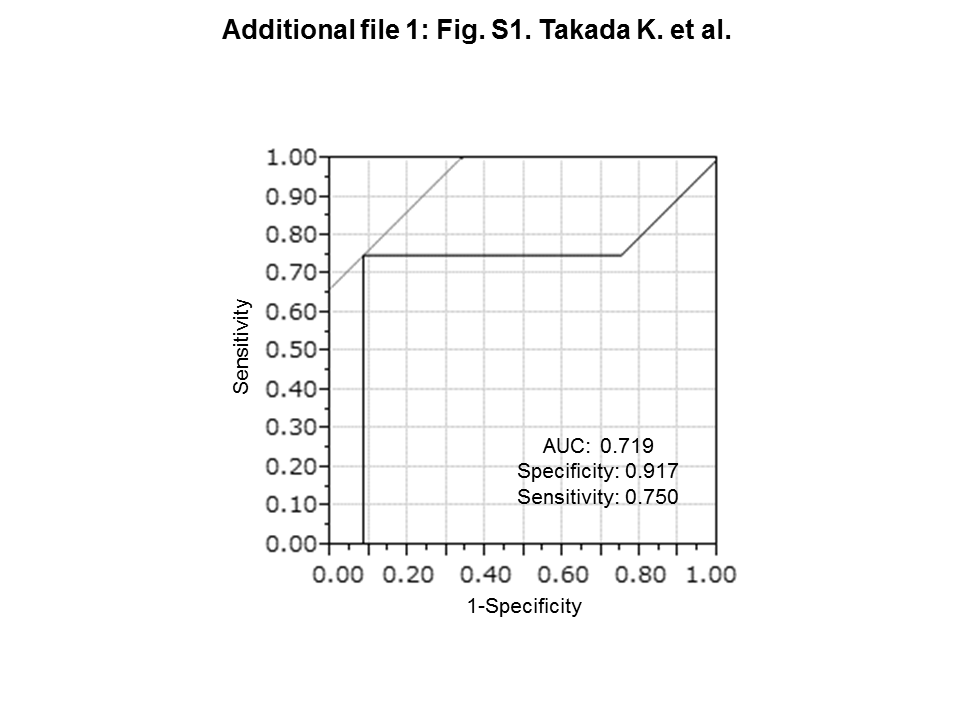

Supplement: Supplementary file 1 — Additional file 1: Fig. S1. The cut-off value of TILs was calculated by receiver operating characteristic (ROC) curve analysis, and the area under the curve (AUC) was 0.719, with a specificity of 0.917 and a sensitivity of 0.750. [file 12967_2018_1692_MOESM1_ESM.tif]
